# Cases of Gun Shot Wounds in the Right Lung, and Their Treatment

**Published:** 1861-02

**Authors:** H. C. Clapp

**Affiliations:** Chicago, Ill.


### CASES OF GUN SHOT WOUNDS OF THE RIGHT LUNG AND THEIR TREATMENT.

BY H. C. CLAPP, M. D., CHICAGO, ILL.

James Clark, resident of Pine Grove township, Van Buren Co., Michigan, a laborer aged 21 years, an Englishman by birth, of a tough, hardy constitution, and sanguine bilious temperament, on the night of the first of August, 1848, together with several others, went on a *charivari* party; the captain being armed with a rifle loaded with powder and a tarred rope wad. While engaged in their sports, the rifle was accidentally discharged, the contents passing through the coat of a Mr. Love, also the coat vest and shirt of the wounded man, entering the right lung at the middle bone of the sternum, breaking off the connections of the second rib from the first bone of the sternum, and the third rib from the second bone, making an opening about the size of an half-dollar, and lodging in the middle lobe of the right lung.

I first saw the patient twelve hours after the accident; found him lying upon the floor, a lady holding a compress closely over the opening. Reaction had not taken place, and whenever the compress was removed, the air and blood would rush with force through the opening.

After making a thorough examination of the wound, I extracted the wad which was deeply imbedded in the lung, together with portions of the powder—burnt lung. Adjusting the sternum and ribs as nearly as possible, I covered the opening with a soft piece of buckskin, with several compresses over that to keep it in its place, and fastened the whole by a roller around the body.

Aug. 3rd, 11 o'clock A. M.—Reaction has thoroughly taken place—pulse full and strong, with considerable fever. The compress has not been removed, no air or blood escapes under it; air only enters the large bronchial tubes of upper lobe of the right lung. He complains of great soreness, but his breathing is less painful than could have been expected from so extensive a wound of the lung. The left lung does not sympathize in the least with the injury of the right one. Bled him 24 oz., ordered a saline cathartic, and that the roller and compress should not be removed, unless suppuration should be so extensive as to impede respiration, in which case remove the roller and compress and let the pus be discharged, and introduce through the opening half a teacupful of chloride of soda (disinfecting solution) to remain for a few minutes, and then to be discharged and the compress and roller replaced as before.

Aug. 4th, 12 M.—Had passed a restless night; compress had to be removed early in the morning, when there was discharged nearly two quarts of dark, bloody, fœtid matter. The cathartic had operated nicely, but still there is too much arterial action; the pulse is 80 per minute, full and hard; complains of great pain in right side. The left lung, as yet, is clear from inflammatory action. Bled him 16 ℥, which produced slight syncope; ordered the disinfecting solution introduced each time the compress was removed for the discharge of the pus.

Aug. 5th, M.—Passed a more comfortable night; the compress had to be removed three times since yesterday noon, and discharged a full pint at each time. Fœtor very offensive, so much so as to be distressing to the patient. The disinfecting solution does not correct the fœtor. Ordered chloride of lime, a large spoonful dissolved in a pint of cold water, and two thirds of a teacupful introduced by the wound after each discharge of matter. The pulse is 100 per minute, with but little fever.

Aug. 6th, M.—Found the patient to-day very weak, and but little inclined to converse; pulse 110, with no fever. Every time the compress is removed, portions of the lung are discharged with the pus. The chloride of lime solution has destroyed the fœtor in a great measure. Continue the solution of chloride of lime as before, and the patient to have a more liberal diet and to be supported by wine three or four times a day.

Aug. 7th—Dr. Andrews, of Paw-Paw, visited the patient with me to-day. Found him very low; pulse 120 per minute, and so weak that he could not be turned over to have the matter discharged freely without great suffering. Several pieces of the lung have been detached and discharged through the opening during the last twenty-four hours. The fœtor has mostly disappeared. Opened the side between the fifth and sixth ribs, to give the matter a free exit. The opening in the side to be kept free by inserting a tent cloth to be removed as often as necessary for the discharge of the pus. Ordered quinine and wine to be given every three hours, together with nourishing diet; solution of chloride of lime to be continued as before.

Aug. 8th—Found patient more comfortable, pulse 112, matter had discharged freely through the opening in the side; the compress had only to be removed to introduce the solution of chloride of lime, on the removal of the compress the air during inspiration does not rush with nearly the force through the opening as at first. Continue treatment.

Aug. 9th—About as yesterday, with but slight if any improvement. Several pieces of the lung have been discharged from the wound. Continue treatment.

Aug. 10th—The pulse is 110, the middle lobe of the lung is wholly destroyed; the lower lobe lies in the cavity slowly wasting away, but the discharge is gradually decreasing in quantity. The left lung begins to enlarge and push itself slowly, but yet perceptibly, to the right side. He has rested better for the last two nights than since the accident. The patient under the same treatment continues gradually to improve from this time on until the first of September, when his pulse is only 80 per minute, and he has but slight fever. The discharge has decreased at least one half. The whole of the middle and lower lobes of the right lung are destroyed, and have been discharged. When the compress is removed from the wound, but a small quantity of air rushes through the opening at each inspiration. By holding a light near the opening you can plainly see into the right cavity of the chest in which was once located the lung which is now wholly destroyed with the exception of a small piece of the upper lobe. The left lung has enlarged to such an extent that nearly one fourth of the opening is closed by it. The solution of chloride of lime to be used hereafter only morning and night; the wine continued three times a day, with liberal nourishing diet.

The patient continued gradually to improve, the discharge became less day by day, and in six months had stopped entirely. The large bronchial tubes had cicatrized and no air passed into the right cavity of the chest. The left lung has enlarged, pushed itself across to the right side. The pleura has attached itself to the edges of the wound and closed the opening entirely. Eleven years have now passed since the accident, and from the time the wound healed to the present, he has been able to attend to his daily labor, and says he feels no inconvenience from the loss of the lung; can do as much labor as previous to the injury, and is not troubled with shortness of breath by hard exercise. The heart is somewhat displaced and pushed to the right side, caused undoubtedly by the enlargement of the left lung.

In this case, I am satisfied the patient owed his recovery in a great measure to the use of the solution of chloride of lime. It not only destroyed the fœtor, but it kept the parts clean, and induced healthy action, which, in my opinion, could not have been obtained from any other remedial agent.

Since the above case, I have used the solution of chloride of lime in several cases of old ill-conditioned ulcers, with the happiest effect. Also in a case of empyema of the left thoracic cavity, of seven years standing, which followed an attack of bilious pneumonia, and was opened about one inch below the left nipple, and for the last three or four years there had formed four fistulous openings from which pus discharged freely. The discharge of matter was so great, that the young man was reduced to a mere skeleton, had slight hectic fever, and was only able to sit up for a short time a day. I opened the side about two inches below the nipple at the lowest fistulous opening, and inserted a silver tube through which I injected a solution of chloride of lime three times a day. Within ten days, the patient showed marked signs of improvement, and in four months was able to resume his trade and labor about half the day at a time, and by the continued use of the solution, the lung and opening in the side have healed entirely. For several weeks after inserting the tube, whenever the cork was withdrawn, at every inspiration, air would rush through the tube. No other remedy was used as an injection into the cavity, but the solution of the chloride of lime.

During the Mormon war in 1846, Mrs. ———, aged twenty-five years, mother of two children, resident of Nauvoo, Hancock county, Illinois, was accidentally shot. I saw the patient one hour after the accident. When the injury occurred, she was leaning over a wash-tub rubbing clothes. The ball struck her just at the lower edge of the scapula, between the sixth and seventh ribs, passed through the right lung and lodged in the right mammary gland. She had bled quite freely from the wound in the back, considerable difficulty in breathing, and whenever her head is raised complains of faintness. Dressed the wound by introducing a tentcloth to be removed if the cavity of the chest became so filled with blood as to impede respiration. I did not extract the ball from the breast. Saw the patient again in six hours; her dyspnœa continued; removed the tentcloth, when there followed from one to one-half pints of fluid blood, which relieved her very much. Reaction does not come on; she is still faint and expectorates some blood. Saw her again the next morning. Found respiration in right lung very painful; reaction not yet fully established. Removed the tentcloth; but little escaped; but on introducing a probe and bearing down the inner flap of the wound, about one pint of dark but fluid blood was discharged with marked relief. She has but slight if any fever; pulse ninety-five; expectorates considerable bloody mucus; ordered a mild cathartic of P. Rhei, and again closed the wound with tentcloth to keep it free until the effusion of blood into the cavity of the chest had ceased. Visited the patient again in the evening; found her easier, respiration better; reaction has taken place; the skin is warm over the whole body; but slight fever; expectorates considerable blood; cathartic has operated; soreness in right side much less than I anticipated from a wound of the lung; saw the patient again next morning; respiration little better; still expectorates some bloody mucus; but less than yesterday. Again removed the tent-cloth and introduced the probe, but there was but a small discharge of sanious pus; pulse 85, with but slight fever. The wound was kept open for two weeks, and was then allowed to heal, and in six weeks she was able to walk about the house. She recovered entirely.

Mr. John Dolby, aged 22 years, of strong constitution and bilious temperament, an Englishman by birth, and resident of Waverley Township, Van Buren County, Michigan, left his home on the 25th day of December, 1857, to visit Paw-Paw on business. While passing by a place where the citizens were gathered for a shooting match, he was shot by the accidental discharge of a rifle. He fell as though shot through the head, but remarked to friends that went to his assistance, that he was badly wounded. Saw the patient four hours after the accident. Found him bolstered up in a large arm-chair, his feet supported by another. The ball, before it struck him, passed through a white-wood board one and one half inch in thickness, and entered the right arm between and above the insertion of the deltoid and pectoralis major muscles, passing below the axilary artery and large nerves, and entering the right side between the second and third ribs, passed through the upper lobe of right lung and in front of the spine, and lodged just under the skin, between the third and fourth ribs, whence I immediately extracted it. In its passage, the ball must have hit the vertebral column, as for several days he had but partial feeling and motion in the lower extremities. His breathing was hard and laborious; skin cold and covered with cold perspiration; pulse feeble and over 100 per minute. Bleeding had been but slight. I introduced a large grooved director through the wound in the arm and side, and there was discharged about eight ounces of blood. Dressed the wound on the back with adhesive plaster, and introduced a long linen tent cloth into the wound through the arm and side, and fastened it by a strip of adhesive plaster. Gave him ½ gr. of morphine; had his feet put into a warm bath, after which sinapisms to be applied to the feet and stomach. The patient to be undressed and placed in bed, a little on left side, with the upper extremities raised, and bottles of warm water placed around the lower extremities to induce reaction.

Saw the patient again on the 26th; pulse 100 and compressible. Reaction comes on slowly, though the skin is warm over the whole body. Respiration difficult and painful; expectorates considerable bloody mucus. Sensation and motion in the lower extremities improving; complains of the greatest pain at the lower part of the right cavity of the chest. Removed the tent-cloth and introduced the grooved director again, which discharged between six and eight ounces of blood from the cavity, with considerable relief. Ordered a cathartic of Epsom salts. Inserted the tent-cloth, with instructions that it should not be removed until the next day.

Dec. 27th—Patient better. His respiration better than yesterday; air enters the whole lung, yet the respiratory murmur is quite dull at the lower lobe, where he complains of very severe pain. Pulse 95, some fever; reaction has fully taken place. The cathartic has operated; feeling and motion in lower extremities rapidly improving. Removed the tent-cloth and inserted the director, when there was discharged from the wound about four ounces of bloody pus. The wound in the back has united by the first intention. Introduced the tent-cloth again. The muscles of the arm and shoulder are somewhat inflammed, and it is very painful to the patient to move the arm.

Dec. 28th—Pulse 90; has some fever and expectorates more bloody pus than yesterday. He complains of the most pain in the right cavity of the chest. Air enters the lower lobe of the lung, showing that respiration is still performed, though somewhat obstructed. On withdrawing the tent-cloth and introducing the director, but a small quantity of blood or pus was discharged. He can use his lower extremities very well, and could sit bolstered up in a chair. Left out the tent-cloth to-day and gave him a teaspoonful of the following mixture, morning, noon and night:

℞. Iodide Potassium, ℥ ss,

Fluid Ext. Sarsaparilla, ℥ vi.

M.

Did not see the patient again until the 30th, when I found him sitting bolstered up in a large arm-chair, smoking his pipe. Respiration much improved; pulse 80 and but very slight fever. Feeling and motion in the lower extremities much improved; expectorates some bloody mucus, but less in quantity. The pain in the right side still continues severe. Opened the wound with the probe, and but little matter escaped. Dressed the wound in the arm with adhesive plaster, and directed the Iodide of Potassium as before.

Visited the patient again on the 3rd of January, 1858, three days after the last visit. Found him improving; pulse 80; less pain in the side. Expectorates but little blood. Feeling and motion in lower extremities still improving; the inflammation of the muscles of the shoulder is subsiding. He feels very much encouraged and thinks he will soon get well.

Visited him on the 6th for the last time. Found his respiration very much improved; pain in the side diminished. The wound through the arm and shoulder had closed. The lower lobe of right lung is dull on percussion; expectoration of blood has entirely ceased. He was carried home on a bed in a sleigh the next day, and I did not see him again until about the 15th of February, when he called on me at my office, in Paw-Paw, having rode fifteen miles in a sleigh that day. Says he still has pain in the right side, and that his breath is shorter than before the accident. He continued to improve, and in the spring was able to resume his labor on the farm.

The above cases are to me very interesting on account of their happy termination and the short time they were each confined, considering the severity of the wounds, and I am satisfied that they show that cases may and do occur where the lung is wounded and bleeding freely, in which the closing of the external wound shuts the only avenue of aid and safety, and the patient dies suffocated from internal extravasation.

